# Supplementary material for: Multiplex lexical networks reveal patterns in early word acquisition in children
Source: Sci Rep. 2017 Apr 24;7:46730. doi: 10.1038/srep46730 (PMC5402256; doi:10.1038/srep46730)
Supplement: Supplementary Information [file srep46730-s1.pdf]

Multiplex lexical networks reveal patterns in early word acquisition in children
Supplementary Information

Massimo Stella, Nicole Beckage and Markus Brede

February 24, 2017

In the following text we provide additional details to the main paper. In Section 1 we give further details about the age of acquisition dataset and characterise the normative ordering. Further technical details on network definitions are given in Section 2. In Section 3 we briefly review the use of single layer network measures referenced in the main text. In the same section we also report and discuss a k-core analysis of the individual MLN layers, in order to explore core-periphery structure of the MLN. Section 4 contains an overview of the multiplex measures adopted in the main text. Section 5 provides some additional analytical results on the random word guessing baseline, which was used as a reference for the overlap word measures introduced in the Methods section of the main paper. Section 6 reports the word gains for many additional ordering experiments carried out and only cited in the main text. Orderings are either based on network features or on word characteristics. Section 7 discusses the optimisation results obtained for betweenness centrality and local clustering, where each layer of the MLN has a different influence. We conclude with Section 8, where we test the influence of individual word features on the predictability outcome of the optimisation procedure.

1 Vocabulary size of children over time

In order to establish an empirical relationship between the number of words learned (i.e. the vocabulary size) and the actual age of children, we considered CDI data collected in a research setting and publicly available [21]. These CDI data exhibit the same trends as the original norming data used in the main text [13] but allow for identification of a child's specific vocabulary size. A linear fitting $y = ax + b$ of the number of words x learned by children of age in months y up to month 30 allowed for us to attribute an average age to children of a given vocabulary size ($a = 0.0151 \pm 0.0006$, $b = 19.7 \pm 0.2$, adjusted R squared $R^2 = 0.53$). Notice that despite individual points in the dataset ranging between ages of 16 up to 30 months, the dependent variable of the linear fit only ranges between 19 and 28 months (cf. SI Fig. S1). This estimated information was used for the plots in Figures 2 and 3 of the main text.

2 Edge definitions

Every node in the MLN represents a word and is replicated across layers. The association and feature norm layers are semantic, in that their relationships provide information about word meanings, while the phonological layer is indeed phonological, i.e. it is based on similarity patterns across word pronunciations. The layer of co-occurrences in child directed speech likely contains information related to semantic, syntactic, and phonological similarity. Notice that the order of layer is not important in our MLN framework.

The free association layer is based on the empirical University of South Florida Free Association Norms [33]. The dataset was built over almost 750,000 empirical free association pairs produced by 6,000 participants as responses to 5,019 stimulus words. Participants were asked to indicate the first target word that came to mind which was related to the presented cue word.

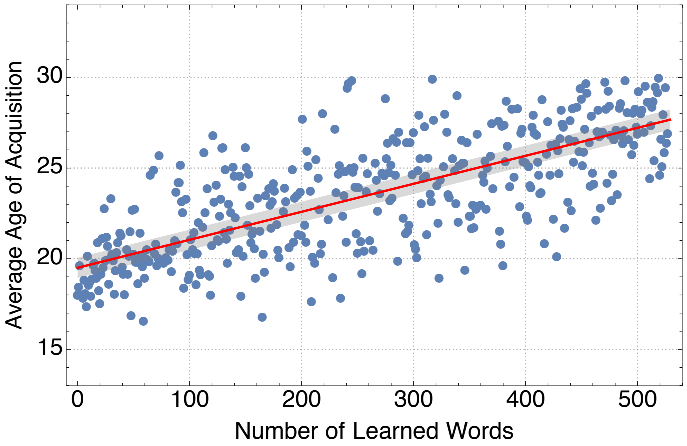

Supplementary Figure 1: Linear fitting $y = ax + b$ of the number of words x learned by children of age in months y up to month 30 from the dataset. The overlay indicates standard errors estimated from the fitting procedure.

The free association norms were obtained by considering only statistically significant associations. From these norms directed edges were retrieved from a cue word (e.g. "eat") to a target word (e.g. "food") with a given normalized frequency (e.g. 0.41). Therefore a weighted directed edge from word A to word B in the original dataset means that "A is freely associated with B". When restricted to the overlap with the CDI vocabulary check list, the network contained 529 words. This CDI association network displayed a reciprocity of 0.38 (i.e. 38% of the edges were bidirectional). However, in this preliminary investigation of multiplex lexical networks, we considered only non-weighted and undirected network layers for the sake of simplicity. We ignored edge weights and converted links from directed to undirected. Hence, in the MLN an edge between words A and B means that one of them is empirically associated with the other (e.g. "food" could be a response to "eat" or "eat" a response to "food"). This undirected network representation used by Steyvers et al. [38] and Hills et al. [25] in their modeling of early language growth using network representations. Instead, a directed version was used in Beckage et al.[5].

The feature norms layer is based on the McRae feature norms dataset [30], i.e. a set of feature norms collected from approximately 725 participants for 541 living (e.g. dog) and non-living (e.g. chair) basic (noun) concepts. Participants were asked to list semantic features of each concept, capturing the most salient and most relevant features. In the semantic features layer generated from this feature norming study, words A and B are connected if they share at least $X = 1$ semantic features (e.g. "balloon" and "ball" share the SHAPE feature).

The co-occurrence layer is based on the CHILDES dataset [29] and it considers word co-occurrences based on child directed speech. In the co-occurrence layer an edge exists between word A and B if word A occurs within five words before or after word B. Because of the high rate of spurious connections, the co-occurrence between two words must have a frequency higher than a given threshold C to be considered as an edge in our graph. We set $C = 45$ in order for the co-occurrence layer to display a connectivity as close as possible to that of the association and feature norms layers. As an example words, "be" and "back" co-occur more than 45 times in the dataset and are therefore connected in the co-occurrence layer of the MLN. From a cognitive perspective, spurious connections are expected in children co-occurrence data because sentences from or directed to children tend to be short and because topics tend to change often [29].

The phonological layer of the MLN is based on phonological similarity among words. We computed similarities based on the IPA phonological word transcriptions obtained from WordNet 3.0 [31]. An edge in the phonological layer between words A and B means that the IPA phonological transcriptions of these words have edit distance one, in agreement with the definition of phonological word similarity adopted in other studies[39]. For instance, the words "bad" and "bat" are phonologically similar, as they differ in their last phoneme, and are therefore connected in the phonological layer.

3 Single-layer network metrics for linguistic networks

All single layers, as defined above have isolated nodes and small connected components, i.e. in each layer some pairs of nodes exists that are not connected by paths. This is evident also from the example visualisation in panel (a) of Fig. 1 from the main text. On the other hand, the whole multiplex lexical network is connected. While the multiplex literature admits several definitions of multiplex connectedness [40, 28, 8], we here adopt the notion of connectedness from De Domenico et al. [19], where a multiplex network is considered connected if its aggregate is connected. As reported in Table 1 of the main text, the largest connected component of the MLN's aggregate network includes all 529 words: the full multiplex lexical network is therefore considered connected.

Notice that the MLN framework considers edge-coloured graphs as multiplex networks following the definition originally proposed within the social sciences [40, 4], where no explicit inter-layer connections are considered. This is a specific case of multiplex networks, which can be generalised in order to consider inter-layer connections as well [28, 8, 18]. In the present MLN representation we ignore costs of jumping across layers, hence no inter-layer edges are considered. This modelling assumption leads to shortest paths over the multiplex structure coinciding with shortest paths over the aggregate network [19]. Thus a shortest path is the network path connecting any two nodes within the smallest number of hops [34].

Shortest path lengths determine, together with clustering, the so-called small-world feature in single-layer networks [41]. We follow established definitions of small-worldness from the literature about cognitive networks [38, 39, 7] according to which a network is a small-world if, compared to random graphs of the same size, it exhibits a significantly higher mean clustering coefficient and a comparable mean shortest path length. In the field of linguistic networks, the small-world feature has been found in semantic networks [32, 35, 26, 38] and phonological networks [39, 36] as well. As evident from the comparison to configuration models in Table 1 from the main text all the layers of the MLN display the small-world feature. As also suggested in previous literature [9, 6, 7, 22], we conjecture that small-worldness may be cognitively beneficial to language learning and use, as it might allow for efficient navigation within semantic memory [22, 9, 3]. Furthermore, empirical evidence has shown that small-worldness is related to language learning in children [6]: semantic network lexicons of late talkers, who are likely to exhibit language processing difficulties, do show small-worldness to a much smaller degree compared to lexicons from children learning words at normative pace.

Small-worlds are defined through the average shortest path length and clustering coefficient. It is worth reminding that the mean clustering coefficient $CC \in [0, 1]$ measures how much neighbourhoods resemble complete graphs. The local clustering coefficient for node i with k_i neighbours and Γ_i edges between its neighbours is:

$$CC_i = \frac{2\Gamma_i}{k_i(k_i - 1)}. \tag{1}$$

As evident from the formula, disconnected nodes and nodes with only one neighbour have an ill-defined local clustering coefficient. In order to exclude them from the mean local clustering $CC = (\sum_i CC_i)/N$, we use the *deformed clustering coefficient* CC_c [27] (see Table 1 from the main text):

$$CC(def.) = \frac{\sum_i CC_i}{N(1 - \theta)}, \tag{2}$$

where θ is the disconnectedness ratio [27], i.e. the fraction of nodes of degree 0 and 1 in the network which by definition cannot contribute to clustering. Previous work [27] highlighted the importance of considering the deformed clustering coefficient when evaluating small-worldness especially when considering small networks (i.e. networks composed of a few hundreds of nodes). This is the motivation for using the deformed clustering coefficient in this work.

The other network measures reported in Table 1 in the main text are: (i) the coefficient of assortative mixing by degree a, measuring degree correlations across network edges, (ii) the fraction of nodes within the largest connected component (*Conn.*) and (iii) the average shortest path length $\langle d \rangle$. Several independent studies identified single-layer linguistic networks as being significantly either more assortative [39, 36, 5] or more disassortative [5] when compared

to random graphs: the assortativity of mixing by degree thus quantifies aspects of structure in language networks that are not present in random graphs. From a cognitive perspective, assortative (disassortative) behaviour indicates the tendency for words having larger numbers of associates/meanings/phonological neighbours (not) to be connected with each other. In the phonological layer this pattern is the consequence of word length correlating with network degree [36, 37]. We conjecture that assortative or disassortative mixing by degree on the semantic MLN layers might be related to a hypothetical navigability through words in the mental lexicon. A higher than random assortative coefficient would express the possibility of reaching a well connected word directly from another well connected word. Instead, disassortative degree mixing, as observed in the association layer, would indicate the possibility of reaching directly specific, less connected terms from broader, more well connected terms.

For a more detailed review of the above network metrics we refer the interested reader to [34].

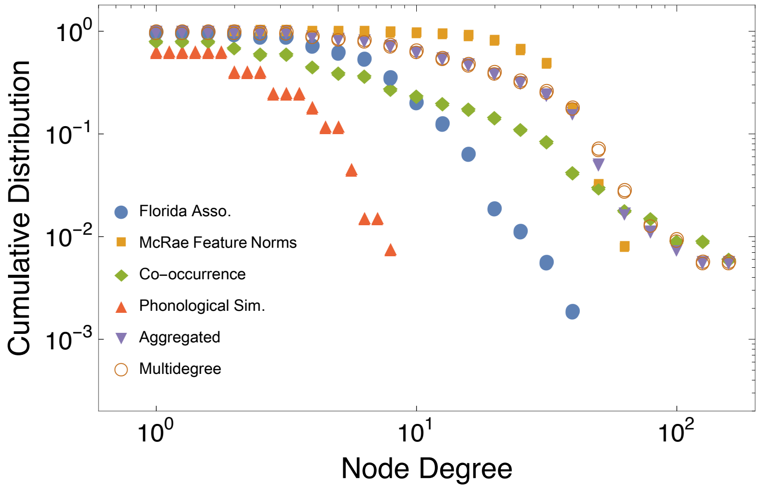

Supplementary Figure 2: Cumulative degree distribution $P(K \geq k)$ of finding a node with individual layer degree or multidegree higher or equal to k within the MLN layers and the aggregate network.

Individual network layers are compared against configuration models, i.e. random graphs with the same degree sequence (and hence the same degree distribution) as the empirical networks. This choice of null model is appropriate, as it rules out effects of the degree sequence on network properties which is important since some of the empirical networks have heavy tailed degree distributions (see SI Fig. 2). Configuration models were sampled by randomising edges while maintaining degree sequences [34]. The resulting network properties, such as clustering coefficient, were tested against analytical estimates [34]. All comparisons of MLN layers in the main text are relative to these configuration models.

3.1 k-core analysis

The high deformed clustering coefficients [27], the relatively high assortativity coefficients, and the relatively flat cumulative degree distributions for degrees up to six (see SI Fig. 2) all suggest the presence of a core-periphery structure in the MLN layers. Notice that words in a densely connected core might have topological features significantly different from those of words in a poorly connected network periphery. Since we relate topological word features to word learning, it is useful to better assess the presence of strongly connected cores within the individual layers of the MLN. For this purpose we perform a k-core analysis [34]. A k-core is a maximal subset of nodes such that each node in the set is connected to at least k others in the same subset. The "maximal" feature indicates that a group of nodes is a k-core if it is not a subset of any larger group that is a k-core. Hence, operatively k-cores can be obtained by repeatedly deleting all nodes of degree less than k in a given network.

In Supplementary Table S1 we list the number of k-cores, while the size of the largest k-core

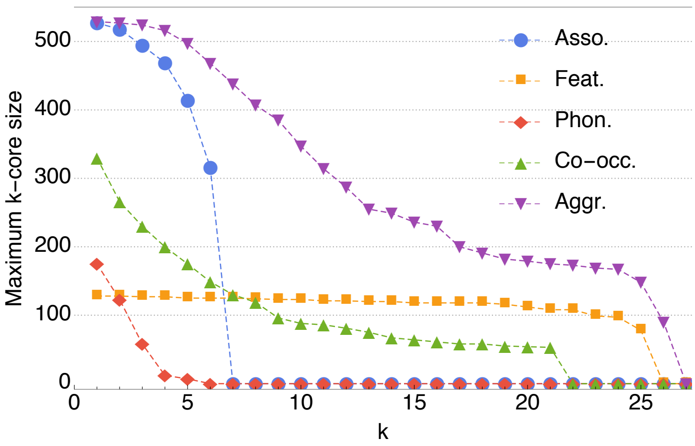

Supplementary Figure 3: Size of the largest k-core for different values of k for the four layers and the aggregate network of the MLN.

for each layer and the aggregated network are visualised in Supplementary Fig. S3. For instance, the phonological network has one largest connected component and 32 other connected components (i.e. k-cores with $k = 1$); these smaller components are also called *linguistic islands* [39]. A high number of linguistic islands, and no highly connected core seems typical for phonological networks [39, 36]. The association layer features a 6-core but no higher k-core. This is further indication that this layer is structurally different from the others, in that it does not display a marked core-periphery structure. In fact, from Table 1 of the main text, the association layer has a bigger largest connected component but comparable average connectivity when compared to the other MLN layers. On the other hand, the feature norms and the co-occurrence layers display k-cores up to $k = 20$, implying the presence of a very densely connected core (i.e. a marked core-periphery structure) in each one of these two layers. Notice that the core from the co-occurrence layer is smaller than the core of the feature norms layer and it also shrinks at a faster rate as k increases. The presence of a very densely connected core in the feature sharing layer is also indicated by the cumulative degree distribution reported in SI Fig. 2. In fact, for $k < 20$ the probability $P(X \geq k)$ of finding nodes with degree equal or higher to k stays nearly at the original value: this indicates the rarity of nodes having degrees $1 \leq k \leq 20$ in the network. Further analysis indicates that the feature norms layer displays up to a 25-core made up of 78 words. The presence of highly connected k-cores is evidence for a rich club effect [34], i.e. highly connected nodes tend to share links among themselves, particularly in the feature sharing layer. This result is in agreement with the high assortative mixing by degree exhibited by this layer and reported in Table 1 of the main text. Also the aggregated network displays k-cores but up to size $k = 26$ and considerably larger than those of the individual layers, again suggesting a rich club effect also when the whole edge-coloured multiplex structure is taken into account.

Interestingly words being part of densely connected k-cores are acquired earlier in the word trajectory based on the empirical age of acquisition. For instance, the 90 words in the 26-core of the aggregate network are ranked 30 ± 1 positions above the average position of ensembles of 90 randomly selected words in the empirical age of acquisition word ranking. Hence, when the normative age of acquisition ordering is considered, words in the densely connected core of the MLN structure are learned earlier than expected at random. This finding corroborates the idea of an interplay between topological features of words in the MLN and patterns in word acquisition. Further, since words in a densely connected core tend to be learned earlier but in general also display higher degree and higher closeness centrality [34], we focus on these two network features in the main text when exploring word acquisition patterns through ordering experiments.

k	1	2	3	4	5	6	7	8	9	10	11	12	13	14	15	16	17	18	19	20
Number of k-cores																				
Asso.	1	1	1	1	1	1	0	0	0	0	0	0	0	0	0	0	0	0	0	0
Feat.	1	1	1	1	1	1	1	1	1	1	1	1	1	1	1	1	1	1	1	1
Co-occ.	1	1	1	1	1	1	1	1	1	1	1	1	1	1	1	1	1	1	1	1
Phon.	33	3	3	4	2	0	0	0	0	0	0	0	0	0	0	0	0	0	0	0
Aggr.	1	1	1	1	1	1	1	1	1	1	1	1	1	1	1	1	1	1	1	1

Supplementary Table 1: Number of k-cores and maximum size of k-cores for k up to 20 in the 4 layers and in the aggregate of the multiplex lexical network.

4 Multiplex network metrics

Multiplex networks are convenient for considering multiple interactions within the same representation [40, 16, 15]. Nonetheless, it is important to quantify whether considering all these interactions jointly is necessary or redundant in terms of topological patterns and interpretation of results. Structural reducibility [16] investigates whether the multiplex paradigm is a suitable model, identifying the presence and extent of redundant topological patterns. In order to assess the benefit of the multiplex representation, we adopt the greedy procedure suggested in [16] and implemented in *muxViz* [17]. This structural reducibility analysis relies on: (i) identifying topologically similar layers, (ii) aggregating layers if appropriate, and (iii) comparing the richness in topological patterns of the aggregated multiplex layers against the aggregated network, obtained by projecting all the edges in the multi-layer structure on a single-layer network.

The procedure identifies similarity of layers and quantifies how distinguishable the multiplex is against aggregate versions of two or more layers. The whole procedure is based on the Von-Neumann entropy of each multiplex layer [16]. This measure is used for determining the richness of topological patterns of each multiplex network aggregation. Let us consider a multiplex network with M layers. In a given aggregation stage σ some of the layers might be aggregated, so that the multiplex network might count $X \leq M$ layers. A relative entropy function q of an aggregation stage σ where X multiplex layers are distinct is computed as:

$$q(\sigma) = 1 - \frac{\sum_{\alpha}^{X} h_\alpha}{X h_A}, \tag{3}$$

where h_α is the Von Neumann entropy of layer α while h_A is the Von Neumann entropy of the aggregated network. The higher the relative entropy q the more distinguishable the multiplex at the aggregation stage a is as compared to the aggregate network. The maximum value of q identifies the aggregation stage that is the most distinguishable compared to the aggregate network. In Fig. 1 (e) of the main text we show that for the MLN the maximum of q is reached when all the layer are kept as separate and no aggregation takes place. This means that the MLN is irreducible, i.e. aggregating any of its layers decreases the information on the topological patterns encapsulated in the multiplex structure. For the mathematical details behind the structural reducibility analysis we refer to [16].

In the performed ranking experiments we adopt single-layer network measures such as degree, closeness and betweenness centralities and their multiplex counterparts. Here we give a brief technical overview over these measures. The *multidegree* [18] or *overlapping degree* [8] m_i of node i is defined as the sum of all the degrees of the node replicas across the $M = 4$ layers of the multiplex:

$$m_i = \sum_{\alpha=1}^{M} k_i^{\alpha}, \tag{4}$$

where $k_i^{(}\alpha)$ is the degree of node i on layer α. The multidegree provides partial information about the connectivity of a node across the multiplex structure and it is one of the simplest and most frequently used measures in the relevant literature [8, 18, 4].

In single-layer networks, the *closeness centrality* c_i measures the distance of node i from all other nodes in the network:

$$c_i = \frac{1}{\sum_j d_{ij}}, \tag{5}$$

where d_{ij} is the shortest distance between nodes i and j. Disconnected nodes have distance infinity and thus do not contribute to the sum. Closeness relates to how fast information is expected to spread from a given node to others in the network. In the main text we consider the generalisation of closeness centrality where multiplex shortest paths are considered (i.e. we allow for "jumps" across layers in order to quantify how close a node is to another one). Notice that this multiplex version of closeness centrality corresponds to the closeness centrality on the aggregate network. In a more general setting one could consider costs for jumps between layers and might wish to introduce more general closeness centrality measures on multiplex networks (cf. [19]). Our choice of identifying multiplex closeness with the closeness of the aggregate is motivated by simplicity of the modelling approach and difficulties of identifying costs of transitioning between layers. This weight of transitions between layers may be related to cognitive functioning but it is a methodologically open question in the literature [1, 42]. Notice that closeness centrality is a problematic measure on disconnected networks [34]. In our case closeness centrality is defined on a fully connected multiplex network, so that biases due to disconnectedness are not present when all the layers are considered at once.

We also consider multiplex shortest paths in the multiplex version of the betweenness centrality b_i which quantifies the extent to which a node lies on the shortest paths between other nodes. For a mathematical definition we refer to [34]. In SI Sect. 6 and 7 we report on the performance of word betweenness for predicting word learning.

Another measure tested in our ordering experiments is the PageRank centrality, which represents the likelihood that a random walker arrives at any particular node navigating a given network by hopping through links. Rather recently *versatile Page-Rank*[20] was introduced as a multiplex generalisation of PageRank, where random walkers can also transition across layers. For the mathematical details behind the formulation of the measure, we refer to [18] and [20]. In SI Sect. 6 we report on the performance of PageRank for predicting word learning.

5 Random word guessing

To evaluate predictions of word acquisition we compare against the reference model of randomly guessing the words to be learned, i.e. the baseline of random permutations of the empirical data set for the word acquisition trajectory. As each word has the same probability of occurring at each position, the probability that a word is correctly guessed as having been learned by time t is $p_w = t/N$. Hence, the average number of correctly guessed words at time t is $\langle n_t \rangle = t^2/N$. Notice that the number of randomly guessed words $\langle n_t \rangle$ approaches the total number of words for larger t, i.e. the word gain measure (see Methods from the main text) becomes less sensitive at later learning stages. The standard deviation σ_t for random word guessing is:

$$\sigma_t = t p_w (1 - p_w) = \frac{t^2 (N - t)}{N^2}. \quad (6)$$

σ_t is one of the three sources of variation affecting ordering experiments presented in the Results section in the main text. The second source is related to shuffling words tied in ranking positions and the third is related to the probabilistic reshuffling of words learned at each month according to normative CDI data. These sources of variation led to error margins on the vocabulary normalised word gains of the order of magnitude of $\approx 10^{-3}$, which are roughly the size of dots reported in Figure 2 from the main text and thus not displayed in that plot for clarity.

Notice that the number of randomly guessed words $\langle n_t \rangle$ represents the expected overlap of random orderings with the normative age of acquisition orderings. In the main text we use $\langle n_t \rangle$ as a reference value for defining both the vocabulary normalised word gain (see the Methods section) and the Z-scores of the overlaps observed through our ordering experiments. We approximate the binomial distribution relative to $\langle n_t \rangle$ and σ_t according to the commonly used constraint that $p_w t$ and $(1 - p_w)t$ have to be both greater than 5 [24]. This implies that we can approximate $\langle n_t \rangle$ and σ_t as coming from a Gaussian distribution when at least $t = 60$ words have been acquired. When valid, this approximation allows for us to use the Z-score statistical testing at a significance level of 5% when at least 60 words have been acquired. The black dashed line in Fig. 2 (b) from the main text marks this region and we adopt it also for indicating the end of the VELS.

6 Tested word orderings and word gains

In the main text we only reported detailed results for word rankings based on a subset of all measures explored. Additional details are given in Supplementary Fig. S4 for word gains for orderings based on degree, word frequency, closeness, word length, betweenness and PageRank. Average word gains are reported on the right end of the plot for each ordering. Notice that the highest word gain is relative to the ordering based on the association degree, which correctly predicts an average of about 19 words as learned on top of the expectation from random guessing. The maximum word gain obtained by the association degree is 42 ± 1 words.

As reported in the main text, adding degrees across different layers together does not guarantee better word gains. In fact the multidegree reaches a lower maximum word gain compared to the association degree only (34 ± 1 vs 42 ± 1). Error bounds are estimated over different normative age of acquisition orderings.

In the main text we tested the ordering based on word frequency computed from the child-directed speech data of the CHILDES dataset [29]. However we computed word frequency on an additional, independently obtained dataset, namely the Opensubtitle dataset [2], which is based on TV subtitles. As reported in Supplementary Fig. S4, the Freq. (Adults) ordering based on Opensubtitle performs much worse (average word gain of 0 ± 2) compared to the frequency counting of the CHILDES dataset (average word gain of 17 ± 2). We conjecture this large gap in performances is due to the different nature of the two frequency datasets. While the CHILDES data set is relative to children speech, the Opensubtitle one is based mainly on TV-series written by and targeted to adults. Unsurprisingly, the lack of predictive power of the adult frequencies suggests that frequency counting from adults is a poor estimator of the word learning dynamics in children.

Word betweenness of the individual layers does perform worse than degree on all the MLN layers. This is not surprising as in all but the association layer several nodes are disconnected and hence have betweenness 0. However, when inter-layer paths are considered, the betweenness centrality computed on the multiplex structure performs noticeably better, with a maximum word gain of 39 ± 2. This finding is in agreement with the structural reducibility analysis from the main text: it supports the importance of considering the whole multiplex structure when investigating patterns among words in the modelled mental lexicon.

Single-layer PageRank for the association layer gives performances close to the degree. This is not a surprise, as PageRank and degree are correlated with each other [34]. However, differently from what happens with the degree, the multiplex version of PageRank for the whole MLN provides predictive power similar to the best single-layer counterpart (i.e. the PageRank in the association layer). This indicates that random walks on the whole MLN are similar to random walks on the association layer, while hub nodes in the association layer might be very different from hub nodes in other layers, so that multidegree and degree in the association layer differ substantially. From a cognitive perspective, the relatively good performance of PageRank confirms previous results about the importance of this measure in exploring the mental lexicon [23].

The word gain was used in the main text for defining the vocabulary normalised word gains (see the Methods section from the main text). A vocabulary normalised word gain of X has to be interpreted as the percentage of words correctly guessed as learned by a given ordering in the whole vocabulary, in addition to those that could be learned at random. For instance, when the vocabulary is made of 100 words, an ordering identifies 24 but 4 would be guessed at random, then the vocabulary normalised word gain would be 0.2 or 20%. Supplementary Tab. S2 reports the average relative word gains for many of the tested orderings.

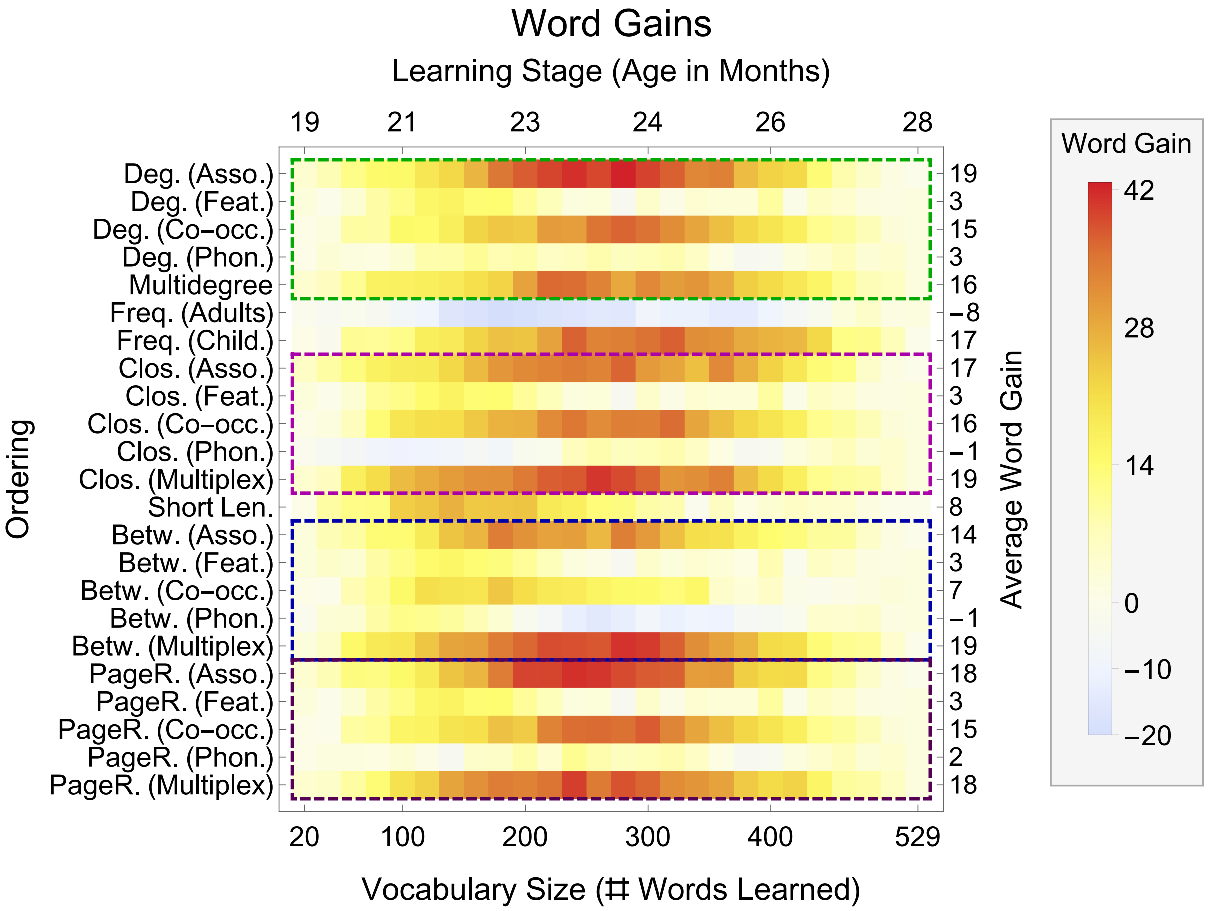

Supplementary Figure 4: Word gains for different orderings at different learning stages. A word gain of 42 means that 42 words were correctly guessed as learned in addition to those that would have been guessed at random. Hence, the gain is considered over random guessing. Word gains averages over the whole learning trajectories are reported on the right. Word orderings are clustered according to their type (e.g. degree based, closeness based, betweenness based, PageRank based). Orderings based on exogenous features are presented as well: Freq. (Adults) comes from the Opensubtitle dataset, Freq. (Child.) comes from the CHILDES dataset, Short. Len. is based on word length.

Ordering	VELS	ELS	LLS
Deg. (Asso.)	0.077	0.13	0.089
Deg. (Feat.)	0.02	0.063	0.01
Deg. (Co−occ.)	0.008	0.089	0.069
Deg. (Phon.)	0.016	0.027	0.011
Multidegree	0.07	0.114	0.073
Freq. (Adults)	−0.006	−0.028	0.003
Freq. (Child.)	−0.006	0.091	0.078
Clos. (Asso.)	0.104	0.138	0.075
Clos. (Feat.)	0.02	0.064	0.01
Clos. (Co−occ.)	0.008	0.113	0.072
Clos. (Phon.)	−0.042	−0.063	0.005
Clos. (Multiplex)	0.07	0.191	0.083
Short Len.	0.045	0.143	0.027
Betw. (Asso.)	0.057	0.122	0.062
Betw. (Feat.)	0.042	0.065	0.009
Betw. (Co−occ.)	0.	0.095	0.03
Betw. (Phon.)	−0.005	0.046	−0.014
Betw. (Multiplex)	0.042	0.163	0.087
PageR. (Asso.)	0.077	0.129	0.087
PageR. (Feat.)	0.02	0.063	0.01
PageR. (Co−occ.)	0.	0.095	0.072
PageR. (Phon.)	0.008	0.009	0.011
PageR. (Multiplex)	0.062	0.144	0.082

Supplementary Table 2: Average vocabulary normalised word gains for different orderings at different learning stages, i.e. the Very Early Learning Stage (VELS), the Early Learning Stage (ELS), the Late Learning Stage (LLS) and the Late Learning Stage (LLS). A vocabulary normalised word gain of 0.112 means that 11.2% words in the vocabulary were correctly guessed as learned by excluding effects due to random guessing. The gain is therefore over random guessing. Word orderings are clustered according to their type (e.g. degree based, closeness based, betweenness based, PageRank based). Orderings based on exogenous features are presented as well: Freq. (Adults) comes from the Opensubtitle dataset, Freq. (Child.) comes from the CDI data, Short. Len. is based on word length.

6.1 Ordering Experiments for the Phonological Layer

Supplementary Fig. S4 and Tab. S2 both demonstrate that the phonological layer does not perform well in terms of predicting the order in which words are learned. In Supplementary Fig. S5 we report the word gain Z-scores (see Methods from the main text) for the two estimators that work best in our analysis, namely word degree and closeness.

When we consider the phonological layer as isolated from the other MLN layers and rank the words according to their degree (closeness) in the phonological layer we obtain orderings whose word gains are compatible with random fluctuations in terms of 2 standard deviations over almost the whole learning trajectory. Hence, within a confidence interval of 95% we can say that degree (closeness) contributions coming from the phonological layer are compatible with random fluctuations. This is why the phonological layer does not perform well in the ordering experiments and in the optimisation experiments as well (see main text).

From a cognitive perspective our technical finding suggests that young toddlers do not use phonological similarities for boosting the likelihood of learning specific new words. This result is supported from recent empirical evidence with children of 18 months of age [14]. As discussed also in the main text, different measures for capturing how similar sounding words are processed by children are required.

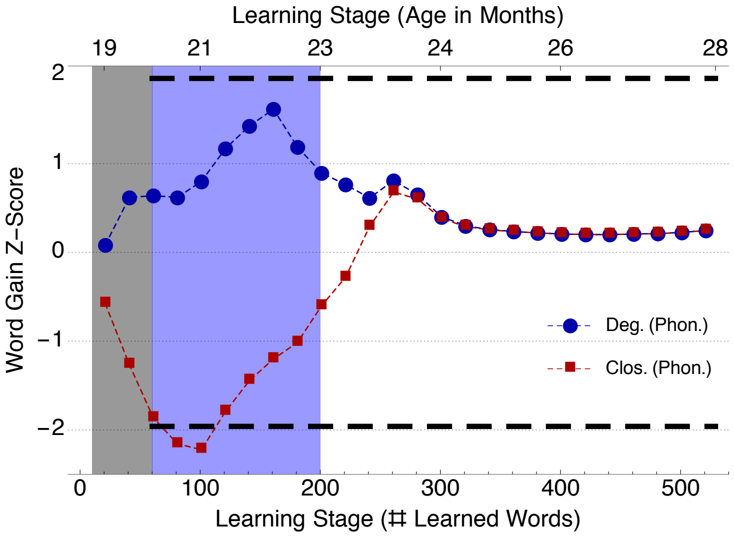

Supplementary Figure 5: Word Gain Z-Score for orderings based on the phonological layer as separate from the other MLN layers. Words are ranked according to either their degree, Deg. (Phon.), or to their closeness, Clos. (Phon.). More connected and closer words are ranked higher. Almost all the retrieved Z Scores are compatible with overlaps provided by random guessing, within the domain of Z score $-1.96 \leq Z \leq 1.96$. Notice that after roughly 300 words are learned the two orderings provide the same average performances because the phonological layer features 268 disconnected words, i.e. words having degree 0 and closeness 1. When ranked, those words for which there is missing topological information become a tie and they are randomised, so that the ranking on that tie is identical to random guessing. We believe this is the technical reason why the phonological layer does not perform well in the optimisation procedure as well.

6.1.1 An Expanded Phonological Layer

Previous literature has found indications that phonological neighbourhood size (i.e. degree in a phonological network) has an impact on word acquisition in young toddles up to 30 months of age [10]. We refer the interested reader to [10] also for a brief review of other works correlating phonology and lexical development in older children.

Based on the optimisation experiments reported in the main text, we cannot interpret the lack of predictive power of the particular phonological layer used in the MLN as a general lack of influence of phonological similarities on lexical acquisition. Instead, this is an indication that

the phonological network induced from the 529 words considered in the study is too small (and thus too fragmented) for providing meaningful information about word acquisition. This finding is not in contrast with the irreducibility analysis: the phonological layer appears to encapsulate different edge patterns compared to other layers. Nonetheless, further analysis with ordering experiments (cf. the previous SI subsection) reveals that these patterns are not more predictive than random guessing for lexical acquisition. In order to test whether the poorly connected topology of the phonological layer is the source of this lack of predictive power we carried out additional experiments explained below.

We took inspiration from the work of [10], where the authors used a phonological network of 12000 words from adult caregivers for testing the influence of phonology on toddlers. Similarly, we considered the larger phonological network from adult native English speakers, already analysed in [36] and including almost 30000 English words. Detailed analysis of the network topology is provided in [36]. The phonological layer of the multiplex analysed in the main paper is a subset of this network. This allows us to evaluate phonological degrees and closeness values of the same 529 words from the MLN on the "extended" topology of the adults' phonological similarities. Based on these two scores we performed additional ordering experiments, which are reported in SI Fig. 6.

We find that the word gains obtained from using either degree or closeness on the extended phonological layer are statistically significant within a 97.5% confidence level during ELS. This indicates that *phonology does indeed play a role on word acquisition rather early on*, compatibly with previous findings from the literature [10]. However, this effect reduces over time and word gains become compatible with random word guessing after 400 words have been learned. A similar result was found in [10], where the influence of phonological word degree on word acquisition vanished around the 30th month of age.

The presence of a statistically significant influence of phonology over word learning motivated us to use the word centralities in the extended phonological network for our optimisation experiments. Results are reported both in the main text (cf. Figure 3) and in SI Sect. 7. Additional analysis relative to the original phonological layer is reported in SI Sect. 8.2.

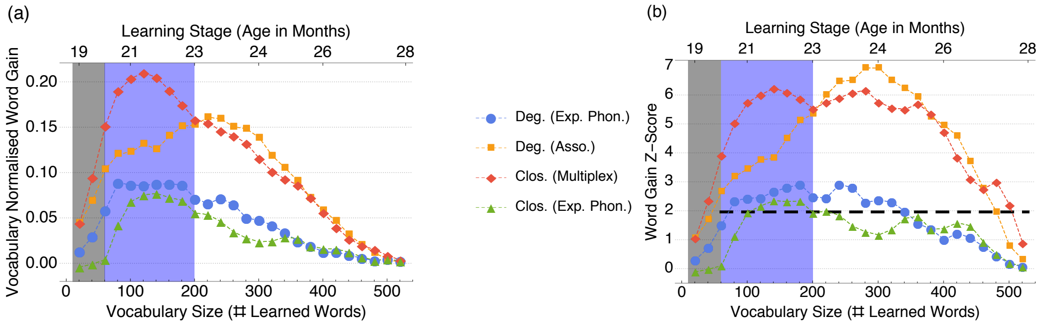

Supplementary Figure 6: Vocabulary normalised word gains (a) and word gain Z-Score (b) for orderings based on the expanded phonological layer (Exp. Phon.) as separate from the other MLN layers. Orderings based on the original MLN association degree and multiplex closeness are reported as well for comparison. The dashed black line in (b) indicates Z-scores equal to 1.96. Both the extended phonological orderings provide predictions above this threshold and hence are statistically significant within a 97.5% confidence level.

6.2 Percentage Word Gains

In order to accompany the results from Fig. 2 of the main text, we introduce also the percentage word gain $P_O(\tau, \omega, t)$, as the difference of word overlaps $O_{aoa}(\tau, t)$ and $O_{aoa}(\omega, t)$ of orderings τ and ω, respectively, normalised by $O_{aoa}(\omega, t)$. We always consider overlaps with the normative age of acquisition orderings. Notice that when ω is a random ordering, then $O_{aoa}(\omega, t) = \langle n_t \rangle$ at time t. The choice of omega can be general. In formulas:

$$P_O(\tau, \omega, t) = \frac{O_{aoa}(\tau, t) - O_{aoa}(\omega, t)}{O_{aoa}(\omega, t)}. \quad (7)$$

The percentage word overlap quantifies the percentage of words that a given ordering provides in comparison to random guessing. For instance, a $P(\tau, \omega, 100) = 50\%$ indicates that when 100 words have been learned, the ordering τ correctly guesses as acquired 50% words more than ordering ω. Results for the case when ω is random ordering are reported in Supplementary Fig. S7. Error bars are estimated from standard deviations of the respective orderings. In VELS and ELS, except for the first point (i.e. when 20 words have been acquired) all our tested orderings display a percentage word gain that is several standard deviations away from the reference value of 0 (Sign Test p-value $< 10^{-5}$ for all the orderings). The highest percentage word gain is the one provided by multiplex closeness centrality, which predicts up to 160% more words compared to random guessing. A percentage word gain against random ordering compatible with zero would imply that the orderings would give improvements compatible with random fluctuations. This implies that all the word gains observed by ranking words according to network features or frequency are statistically not compatible with random fluctuations when at least 40 words are learned. This finding supports results coming from the Z-scores presented in the main text. Notice that while P_O allows for a statistical assessment of the significance of word gains in VELS (something that Z-scores cannot do), the percentage overlap P_O suffers from approaching the 0 reference level for all the orderings at the middle of LLS. In this phase, the Z-scores provide a clearer picture of the predictability of different orderings (see Fig. 2 in the main text). Notice that the Z-scores provide a more robust statistical testing of our findings.

Notice that we consider also another case when ω is the association degree ordering and results are reported in Supplementary Fig. S8. The multiplex closeness performs up to 25% better in ELS than association degree and the difference in performance between these two orderings is several standard deviations away from 0 (Sign Test p-value $< 10^{-5}$ for all the orderings). The unmatched predictive power of closeness centrality is what marks the whole early learning stage. At later stages, during LLS, the multiplex closeness and the association degree perform similarly. Interestingly, frequency performs worse than either multiplex closeness or association degree during early learning stages. Word length and multiplex PageRank perform only up to 10% better than association degree in ELS. The percentage word overlap confirms the claims from the main text that multiplex closeness outmatches all the other considered orderings during early learning stages.

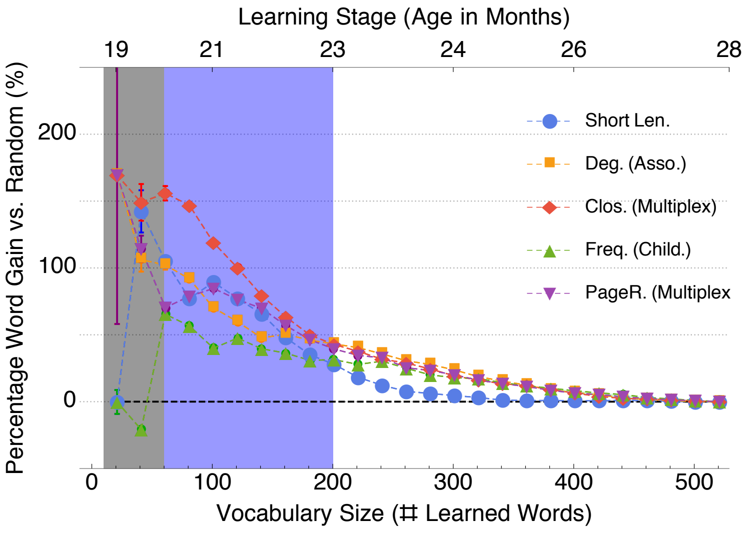

Supplementary Figure 7: Percentage word gains against random guessing for the word orderings reproduced in Figure 2 of the main text. A percentage word gain of 100% at a given learning stage means that a word ordering leads to guessing 100% words more compared to random guessing. Error bars are based on standard deviations. All the orderings in VELS provide percentage word gains that are clearly away from 0 (Sign Test p-value $< 10^{-5}$ for all the orderings).

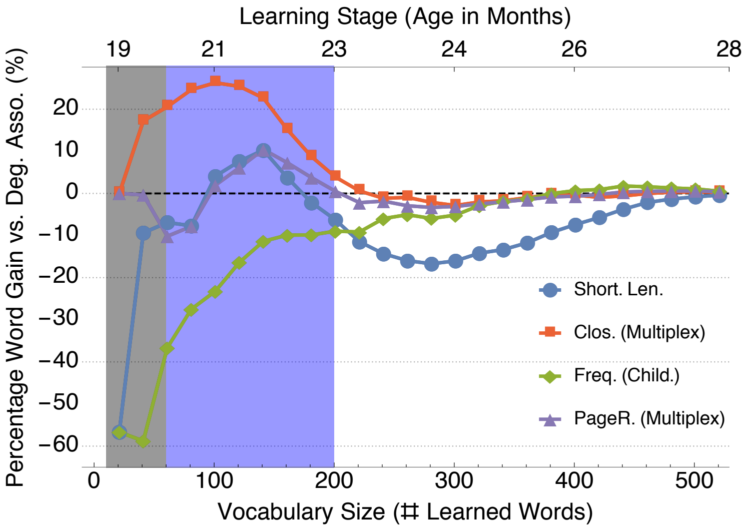

Supplementary Figure 8: Percentage word gains against the association degree ordering for the word orderings reproduced in Figure 3 of the main text. A percentage word gain of 20% at a given learning stage means that a word ordering leads to guessing 20% words more compared to association degree. Error bars are based on standard deviations and are the same size of the dots. The peak in ELS for the multiplex closeness is clearly incompatible with a 0 difference (Sign Test p-value $< 10^{-5}$ for all the orderings).

6.3 Influence of taxonomic relationships on word gains

Many English words refer to categories that are taxonomically organized, e.g. "horse" is a type of "animal". This taxonomic organisation results into basic, super-ordinate and sub-ordinate level object categories. Super-ordinate categories have broader semantic fields than sub-ordinate level categories. This taxonomic organization can influence the semantic relationships giving rise to free associations, feature sharing norms and co-occurrences we represented in our multiplex lexical network.

Hence, explicitly quantifying how word features in individual multiplex layers correlate with a superordinate and subordinate categorisation of words in the children's vocabulary might be of relevance for understanding the mechanisms behind word acquisition in young children.

We focus here on quantifying how the taxonomic word organisation acts as a mediator variable correlating with the two most predictive word features in our framework: degree of words in the free associations and the closeness of words on the multiplex structure.

We retrieved from WordNet 3.0 (the version curated by Wolfram Research) a network of hyponymy relationships, where nodes represent words and the directed edge $A \rightarrow B$ means that A is a type of B. This hyponymy network included roughly 350000 English words. The in-degree of a node is defined as the number of directed edges pointing to that node. In this hyponym network words with higher in-degree represent broader words, as they have more words that fall into their semantic category. For instance, considering the edges "$pigeon \rightarrow bird$" and "$dove \rightarrow bird$", then bird would have indegree 2 while pigeon and dove would have indegree 0. We would then consider "bird" as being a broader term than either "pigeon" or "dove".

We assume that in-degree on the whole hyponym network is a good proxy of the mediator variable that distinguishes super-ordinate words (high indegree) from sub-ordinate (low indegree) in the English language.

We then correlated multiplex closeness and association degree of MLN words with their in-degree in the whole hyponymy network. We used the Kendall Tau to quantify correlations. Results indicate that the MLN association degree correlates with in-degree almost three times more compared to multiplex closeness (Kendall Tau of association degree ≈ 0.20, p-value $< 10^{-5}$ vs Kendall Tau of multiplex closeness ≈ 0.07, p-value < 0.01).

The different Kendall Taus indicate that the association degree is a better proxy for detecting superordinate words (i.e. words with higher indegree in our case) compared to closeness

centrality.

As discussed in the main text, we conjecture that the association degree does not perform well in VELS and ELS because it would tend to guess more super-ordinate words than multiplex closeness would. Norming studies suggest [25] that children tend to learn general level categories before super- or subordinate classes (e.g. "dog" is learned before "animal", "chair" before furniture), a fact that is not captured well by the association degree ordering.

This is compatible with what we observe in our optimisation experiments (see the new Fig. 3, panels (c) and (d)), where the association layer contributes the most to optimised orderings using both degree and closeness but only outside of VELS. This network pattern suggests the emergence of a boosting effect in learning the generalisations of words previously acquired during VELS and ELS. There generalisations represent super-ordinate words that are captured predominantly by the association layer and not by the others (otherwise we would expect for all the layers to keep the same influence after VELS and ELS).

7 Optimisation Experiments

In Supplementary Tab. S3 we report the average vocabulary normalised word gains for the optimal orderings obtained by combining degrees and closeness centralities. Further, in Supplementary Fig. S9 we report the word gain Z-scores for the optimal orderings obtained by combining degrees and closeness centralities. These results complement the visualisation in panels (a) and (b) of Fig. 3 in the main text. In combination they demonstrate that not even the optimal combination of word degrees can perform better than multiplex closeness in terms of word predictability. Also, differently from the ordering experiments, for the optimised trajectories the Z-scores at the end of VELS are now clearly statistically significant. Ternary plots in panels (a) and (b) in SI Fig. S10 visualise the optimisation landscape of vocabulary normalised word gains over the remaining three layers at the end of VELS, middle of ELS, and middle of LLS. The region of optimal word gains tends to narrow and changes its location in the landscape over time, corroborating the idea that different linguistic information plays different roles at different stages of development.

Very similar results from SI Fig. S9 were obtained for the MLN with the extended phonological layer, for which the relative word gains are reported in SI Tab. S3 while the optimisation results are reported in SI Fig. S11.

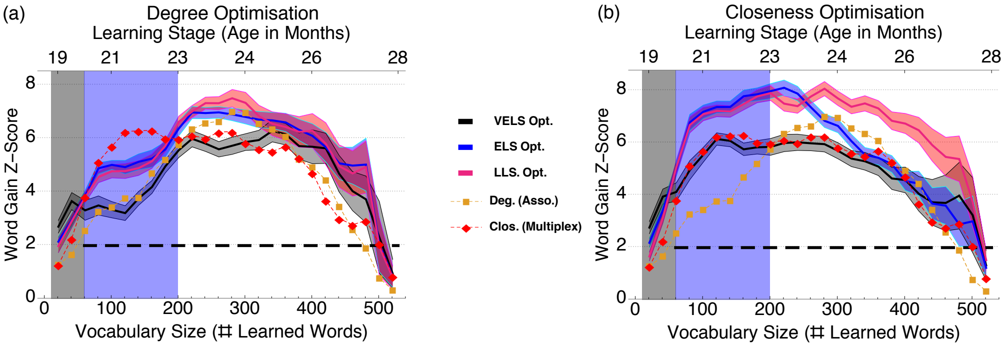

Supplementary Figure 9: Word gain Z-scores for: (a) the optimal orderings of degree and (b) for closeness. The ordering experiments based on multiplex closeness and on association degree are reported as dots. The dashed black line indicates the range of words when the Z-Scores can be approximated to normal and it identifies the 95% confidence level (Z=1.96). Error bars are based on standard deviations from the optimised ensembles of word trajectories. In panel (a), the peak in ELS for the multiplex closeness is clearly larger than the peak for optimal combinations of word degrees (Sign Test p-value $< 10^{-5}$).

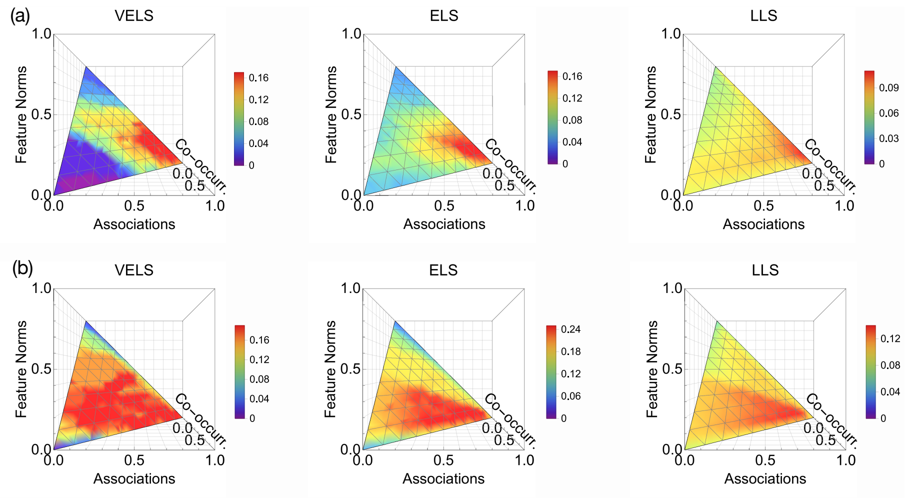

Supplementary Figure 10: Ternary plots of the average relative word gains at the end of VELS, the middle of ELS and the middle of LLS for degree (a) and closeness (b) optimisation. Optimisation results relative to these ternary plots are reported in Figure 3 of the main text.

7.1 Linear optimisation of betweenness and local clustering

Since betweenness on the whole MLN performs slightly worse than association degree, as reported in SI Sect. 6, we also used the betweenness centralities of words within the MLN layers as a basis for scores to calculate optimal combinations of layers. We considered local deformed clustering as well, as defined in SI Sect. 3. We chose these two additional network features because they utilise the multiplex MLN structure at global and local levels.

When betweenness was used, the retrieved optimal layer importances did not change over the VELS, ELS or LLS stages. Therefore only one relative word gain curve is reported in Supplementary Tab. S3. Predictability results coming from the optimal linear combination of betweenness centralities are overall inferior to the ordering results with multiplex closeness and the single-layer association degree. Betweenness optimisation leads to similar results compared to the ordering experiments with multiplex betweenness (see Supplementary Tab. S2). The fact that betweenness provides lower predictability power than closeness on the whole MLN suggests that early learned words have higher closeness but lower betweenness. Hence they are words closer to others on the MLN structure but they are not necessarily part of many shortest paths as the MLN topology offers different short-cuts for navigating through words.

In the main text we reported the results of optimisation procedures based (i) on a local network statistics such as degree and (ii) on a global network statistics such as closeness centrality. Between the global and local extremes, we also investigated the optimisation of a second-order local network statistics such as the local clustering coefficient. Predictability results are reported in Supplementary Tab. S3. The optimisation over local clustering performs worse than the empirical multiplex closeness centrality and the linear optimisation results for both degree and closeness. This suggests further that the multiplex structure is supportive in capturing normative language learning trajectories of young children.

The inferior predictability performances of local clustering and betweenness, particularly in ELS, support the idea reported in the main text that closeness centrality, and therefore network distances, capture word correlations that are fundamental and relevant to normative word learning in young children. The idea that network distances are relevant for the cognitive processes regulating the ML is supported by previous experimental evidence [12, 11, 32].

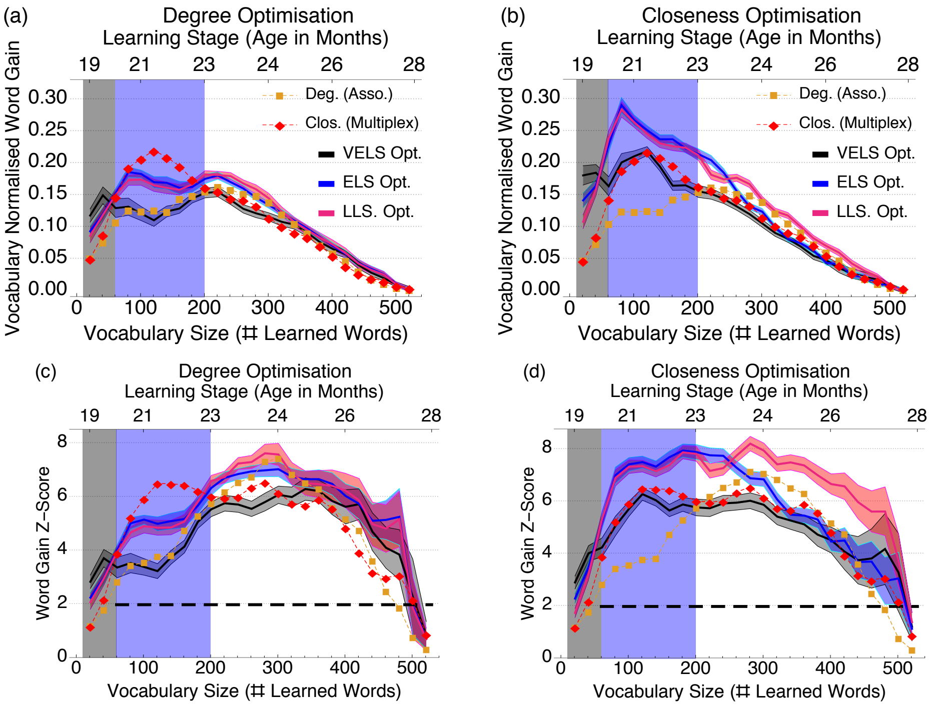

Supplementary Figure 11: Optimisation results for degree (a) and closeness (b) in the networked lexicon with the extended phonological layer. Word gain Z-scores for (a) the optimal orderings of degree and (b) for closeness in the networked lexicon with the extended phonological layer. The ordering experiments based on multiplex closeness and on association degree are reported as dots. The dashed black line indicates the range of words when the Z-Scores can be approximated to normal and it identifies the 95% confidence level (Z=1.96). Error bars are based on standard deviations from the optimised ensembles of word trajectories.

7.2 Non-linear optimisations

Also non-linear combinations of word scores across layers were explored and investigated. In particular, we tested convex combinations of the type:

$$s_w = \alpha \frac{(s_w^{(Asso)})^a}{\sum_w (s_w^{(Asso)})^a} + \beta \frac{(s_w^{(Feat)})^b}{\sum_w (s_w^{(Feat)})^b} + \gamma \frac{(s_w^{(Co-occ)})^c}{\sum_w (s_w^{(Co-occ)})^c} + (1-\alpha-\beta-\gamma) \frac{(s_w^{(Phon)})^{3-a-b-c}}{\sum_w (s_w^{(Phon)})^{3-a-b-c}}, \quad (8)$$

where the coefficients $\alpha, ..., \gamma$ give layer influences, and the exponents $a, ..., c$ allow for non-linear influences of the various layers. Results are reported in Supplementary Tab. S3; we note that including non-linear influences only gives marginal corrections to the linear model explored in the main paper. Importantly, the main findings about the particular role of closeness centrality remains unchanged.

8 Shuffling Models

8.1 Random shuffling models for the optimisation procedure

In this section we test how correlations of closeness and degree centralities among layers in the MLN influence results of optimisation procedure. In particular, we aim to test of prediction accuracies of the same quality as reported in the main text could be obtained from (multiplex) networks in which correlations have been destroyed. We consider null models for multiplex structure by shuffling the multiplex network, i.e.:

1. A multiplex network made of layers having an unchanged topology but randomly shuffled node labels. We call this the Global Label-Shuffled null model (GLS), and essentially for

Ordering	VELS	ELS	LLS	Total
Deg. (Asso.)	0.08(1)	0.13(1)	0.09(1)	0.09(1)
Clos. (Multiplex)	0.07(1)	0.19(1)	0.08(1)	0.10(1)
Opt. Deg. (VELS)	0.16(4)	0.12(2)	0.08(1)	0.09(1)
Opt. Deg. (ELS)	0.14(2)	0.17(2)	0.09(1)	0.11(1)
Opt. Deg. (LLS)	0.14(2)	0.17(2)	0.08(1)	0.11(1)
Opt. Clos. (VELS)	0.19(3)	0.19(2)	0.07(1)	0.12(2)
Opt. Clos. (ELS)	0.16(2)	0.25(2)	0.09(1)	0.13(1)
Opt. Clos. (LLS)	0.14(2)	0.24(2)	0.10(1)	0.14(1)
Opt. Deg. Ext. (VELS)	0.17(4)	0.12(2)	0.09(1)	0.10(1)
Opt. Deg. Ext. (ELS)	0.15(2)	0.17(2)	0.10(1)	0.12(1)
Opt. Deg. Ext. (LLS)	0.15(2)	0.17(2)	0.09(1)	0.11(1)
Opt. Clos. Ext. (VELS)	0.19(3)	0.20(2)	0.08(1)	0.13(2)
Opt. Clos. Ext. (ELS)	0.16(2)	0.26(2)	0.09(1)	0.13(1)
Opt. Clos. Ext. (LLS)	0.14(2)	0.25(2)	0.10(1)	0.14(1)
Opt. Betweenness	0.11(2)	0.15(2)	0.08(1)	0.09(1)
Opt. Loc. Clus. (VELS)	0.10(2)	0.13(2)	0.06(2)	0.08(2)
Opt. Loc. Clus. (ELS)	0.10(2)	0.16(2)	0.08(2)	0.09(1)
Opt. Loc. Clus. (LLS)	0.10(2)	0.16(2)	0.07(2)	0.09(1)
Nonl. Opt. Deg. (VELS)	0.18(2)	0.13(2)	0.09(1)	0.10(1)
Nonl. Opt. Deg. (ELS)	0.15(2)	0.17(2)	0.09(1)	0.11(1)
Nonl. Opt. Deg. (LLS)	0.13(2)	0.17(2)	0.10(1)	0.11(1)
Nonl. Opt. Clos. (VELS)	0.19(3)	0.20(2)	0.08(1)	0.12(2)
Nonl. Opt. Clos. (ELS)	0.16(2)	0.25(2)	0.10(1)	0.13(1)
Nonl. Opt. Clos. (LLS)	0.15(2)	0.24(2)	0.11(1)	0.15(1)

Supplementary Table 3: Optimised vocabulary normalised average word gains for the empirical degree in the association layer and multiplex closeness centrality (top), the optimal degrees and closeness for the whole MLN (middle - Opt.), the optimal degrees and closeness with the extended phonological layer (middle - Ext.), the optimal betweenness and local clustering linear combinations (middle) and the non-linear combinations of degree and closeness centralities, respectively (bottom). Entries are averaged while: 40 words have been learned in VELS, 160 words have been learned in ELS, when 369 have been learned in LLS and when all the 529 words have been learned (Total). Parentheses indicate error bounds as standard deviations: 0.14(2) indicates 0.14 ± 0.02.

Ordering	VELS	ELS	LLS	Total
Opt. Deg. (VELS)	0.16(4)	0.12(2)	0.08(1)	0.09(1)
Opt. Deg. (ELS)	0.14(2)	0.17(2)	0.09(1)	0.11(1)
Opt. Deg. (LLS)	0.14(2)	0.17(2)	0.08(1)	0.11(1)
GLS Opt. Deg. (VELS)	0.03(2)	0.02(1)	0.01(1)	0.02(1)
GLS Opt. Deg. (ELS)	0.02(2)	0.03(1)	0.01(1)	0.02(1)
GLS Opt. Deg. (LLS)	0.02(2)	0.03(1)	0.01(1)	0.02(1)
ILS Opt. Deg. (VELS)	0.002(2)	0.002(2)	0.001(1)	0.002(2)
ILS Opt. Deg. (ELS)	0.001(1)	0.003(2)	0.002(2)	0.002(2)
ILS Opt. Deg. (LLS)	0.001(1)	0.003(2)	0.002(2)	0.002(2)
Opt. Clos. (VELS)	0.19(3)	0.19(2)	0.07(1)	0.12(2)
Opt. Clos. (ELS)	0.16(2)	0.25(2)	0.09(1)	0.13(1)
Opt. Clos. (LLS)	0.14(2)	0.24(2)	0.10(1)	0.14(1)
GLS Opt. Clos. (VELS)	0.03(3)	0.03(2)	0.01(1)	0.02(2)
GLS Opt. Clos. (ELS)	0.03(2)	0.04(2)	0.01(1)	0.03(2)
GLS Opt. Clos. (LLS)	0.02(2)	0.04(2)	0.01(1)	0.03(1)
ILS Opt. Clos. (VELS)	0.002(3)	0.002(2)	0.001(1)	0.002(2)
ILS Opt. Clos. (ELS)	0.001(2)	0.003(2)	0.001(1)	0.001(1)
ILS Opt. Clos. (LLS)	0.001(2)	0.003(2)	0.001(1)	0.001(1)

Supplementary Table 4: Average vocabulary normalised word gain obtained when optimising degree (Deg.) or closeness centrality (Clos.) from the empirical dataset, the shuffled GLS model and the shuffled ILS model. The results were averaged over 500 shuffles. Error bars represent standard deviations and are denoted within parentheses, i.e. 0.19(3) means 0.19 ± 0.3.

each network metric every word receives a word score randomly chosen from the word scores of the original empirical multiplex network. This gives word scores $s_w^{(GLS)}{=}s_{\Pi}(w)$ where Π indicates a random permutation of word labels.

2. A multiplex network preserving the topology of the empirical layers but with re-shuffled node labels in each layer. We call this the Independent Label-Shuffled null model (ILS). In this model associations of words between layers are randomised, i.e. replicas of a node on different layers can be associated with different topological identities of words in the original multiplex network. This results in word scores $s_w^{(ILS)} = \alpha s_{\Pi_1(w)}^{(asso)} + \beta s_{\Pi_2(w)}^{(feat)} + (1 - \alpha - \beta) s_{\Pi_3(w)}^{(co-occ)}$, where Π_1, Π_2, Π_3 indicate potentially different random permutations of the word list.

It is important to note the following:

- The Global Label-Shuffled (GLS) null model preserves the underlying distribution of global scores $\{S_i\}$ at a global network level, hence the name. However, it does not preserves the local multidegree or multi-closeness centralities of a given node (i.e. on the microscopic level). This model is a suitable null model because, in addition to the above global preservation of the scores, it also preserves inter-layer degree/closeness correlations (i.e. a hub in one layer might tend to be a poorly connected node in another layer).
- The Independent Label-Shuffled null model does not preserve the underlying distribution of global scores, since it reallocates node labels independently at random on each layer. This model preserves only the distributions of intra-layer scores $\{\{s_i^{(asso)}\}, \{s_i^{(feat)}\}, \{s_i^{(co-occ)}\}\}$. It disrupts inter- layer correlations.

Using these null multiplex models, we estimate the word gains. Results from Supplementary Tab. S4 show that:

1. Even when the global distributions of scores are preserved, the optimisation procedure does not lead to results close to the optimal results for the non shuffled multiplex network, both results are separated by at least a factor of six.

2. Results for the ILS are compatible with a random ordering when error bars are considered. Maintaining the distributions of scores in each individual layer is not enough to guarantee better results than random word guessing.

3. Preserving the inter-layer multiplex correlations (as in the GLS null model) is fundamental in providing better performances of the optimisation procedure.

All in all, prediction on shuffled multiplex networks are substantially less accurate than predictions reported in the main paper. This gives further support for the main findings of the paper, i.e. correlations on the (multiplex) are an important determinant in word acquisition.

8.2 Importance testing for the phonological layer

Previous literature has reported correlations between degrees in the phonological layer and word learning in young children (cf. [10]). In contrast, the main paper does not find a significant influence of phonological information on word acquisition. The purpose of this section is to demonstrate that in principle our methodology can discover and appropriately quantify such correlations if present in the dataset. To demonstrate this, we re-allocate word labels on the phonological layer in such a way that phonological degrees correlate with acquisition orderings to a tunable extent. This can be achieved by reshuffling randomly selected words in a directed manner until a desired correlation level has been achieved. Then we run optimisations for a multiplex composed of unaltered semantic layers and the reordered phonological layer. We randomise over 20 probabilistic age of acquisition orderings and perform 10 Monte Carlo robustness samplings for each age of acquisition ordering. We consider three hypothetical scenarios: a situation in which phonological degree and age of acquisition have a small correlation (Kendall Tau of 0.1), a situation with intermediate strength correlations (Kendall Tau 0.3) and a situation with very high correlations (Kendall Tau 0.8). Notice that the degree-based ranking of the original phonological layer correlated with the normative age of acquisition with a Kendall Tau of ≈ 0.01. Results of the reordering experiments are given in Figure SI Fig. 12.

In agreement with expectations, we observe that the optimisation assigns: (i) small but non-negligible weight to the phonological layer for the small correlation scenario, (ii) significant weight to the phonological layer for intermediate strength correlations and (iii) dominant weight to the phonological layer in the large correlation scenario. As shown in the main paper, in particular also the association layer correlates with intermediate strength with age of acquisition (Kendall Tau ≈ 0.24). Hence, in particular in the intermediate correlation scenario in which correlations of the phonological and of the association layer with age of acquisition are of similar magnitude, we observe interactions between these two layers, i.e. the phonological layer first dominates and then loses relative weight when the association layer gains influence. This effect is also detectable for the large correlation scenario, but here correlations in the phonological layer are always dominant.

These experiments clearly demonstrate that our methodology is able to attribute layer weightings according to the predictive power of layers for word acquisition. In particular, the methodology is in principle capable of also quantifying correlations in the phonological layer, that is, the observation of phonological layer weights in the order of 10^{-3} is not to be attributed to the methodology but rather to the lack of predictive power encapsulated within network statistics of the layer. Compared to the extended phonological layer we used in SI Sect. 6.1.1, the original smaller layer is much less connected and more fragmented. Both the layers are based on the same phonological similarity measure but the extended phonological layer includes more than 29000 words, while the one originally used in the MLN includes only 529 words. We therefore consider the lack of performance of the MLN phonological layer to be a matter of quantity (rather than quality).

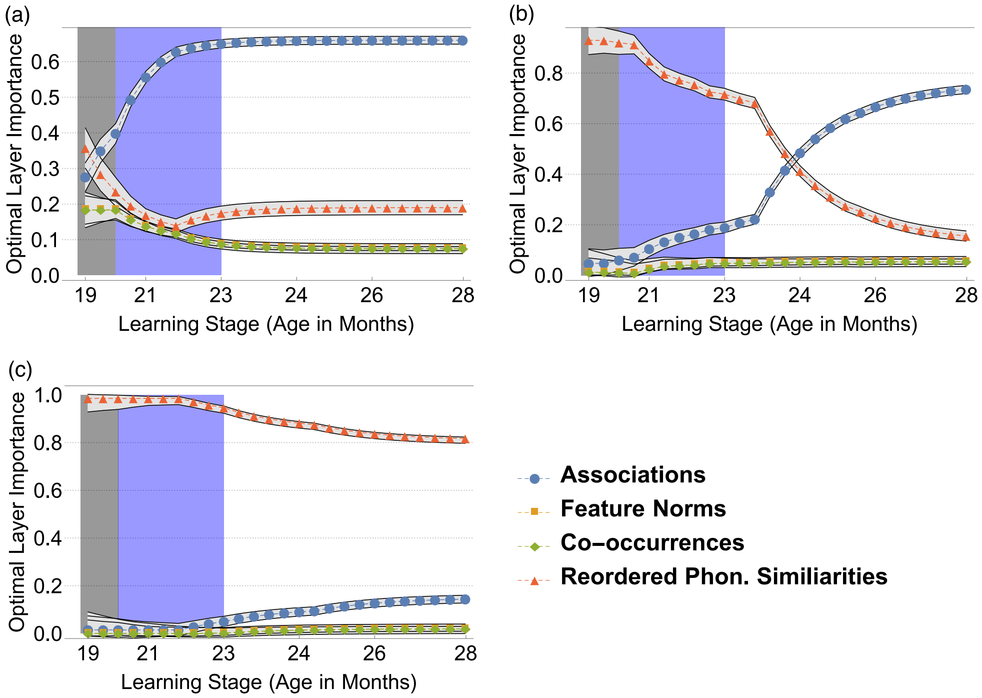

Supplementary Figure 12: Optimal layer weights obtained from the degree optimisation where the labels on the phonological layer are reshuffled in order for word degrees to correlate with the age of acquisition ordering according to a Kendall Tau of 0.1 (top left), 0.3 (top right) and 0.8 (top left).

References

[1] Jean Aitchison. *Words in the mind: An introduction to the mental lexicon.* John Wiley & Sons, 2012.

[2] Adrien Barbaresi. *Language-classified Open Subtitles (LACLOS): download, extraction, and quality assessment.* PhD thesis, BBAW, 2014.

[3] Andrea Baronchelli, Ramon Ferrer-i Cancho, Romualdo Pastor-Satorras, Nick Chater, and Morten H Christiansen. Networks in cognitive science. *Trends in cognitive sciences*, 17(7):348–360, 2013.

[4] Federico Battiston, Vincenzo Nicosia, and Vito Latora. Structural measures for multiplex networks. *Physical Review E*, 89(3):032804, 2014.

[5] N M Beckage, A Aguilar, and E Colunga. Modeling lexical acquisition through networks. *Proc. of the 37th Conf. of the Cog. Sci. Society*, 2015.

[6] Nicole Beckage, Linda Smith, and Thomas Hills. Small worlds and semantic network growth in typical and late talkers. *PloS one*, 6(5):e19348, 2011.

[7] Nicole M Beckage and Eliana Colunga. Language networks as models of cognition: Understanding cognition through language. In *Towards a Theoretical Framework for Analyzing Complex Linguistic Networks*, pages 3–30. Springer, 2015.

[8] Stefano Boccaletti, G Bianconi, R Criado, Charo I Del Genio, J Gómez-Gardeñes, M Romance, I Sendina-Nadal, Z Wang, and M Zanin. The structure and dynamics of multilayer networks. *Physics Reports*, 544(1):1–122, 2014.

[9] Javier Borge-Holthoefer and Alex Arenas. Semantic networks: structure and dynamics. *Entropy*, 12(5):1264–1302, 2010.

[10] Matthew T Carlson, Morgan Sonderegger, and Max Bane. How children explore the phonological network in child-directed speech: A survival analysis of children's first word productions. *Journal of memory and language*, 75:159–180, 2014.

[11] Allan M Collins and Elizabeth F Loftus. A spreading-activation theory of semantic processing. *Psychological review*, 82(6):407, 1975.

[12] Allan M Collins and M Ross Quillian. Retrieval time from semantic memory. *Journal of verbal learning and verbal behavior*, 8(2):240–247, 1969.

[13] P S Dale and L Fenson. Lexical development norms for young children. *Behavior Research Methods, Instruments, & Computers*, 28(1):125–127, 1996.

[14] Isabelle Dautriche, Daniel Swingley, and Anne Christophe. Learning novel phonological neighbors: Syntactic category matters. *Cognition*, 143:77–86, 2015.

[15] Manlio De Domenico, Clara Granell, Mason A Porter, and Alex Arenas. The physics of spreading processed in multilayer networks. *Nature Physics*, 12:901–906, 2016.

[16] Manlio De Domenico, Vincenzo Nicosia, Alexandre Arenas, and Vito Latora. Structural reducibility of multilayer networks. *Nature communications*, 6, 2015.

[17] Manlio De Domenico, Mason A Porter, and Alex Arenas. Muxviz: a tool for multilayer analysis and visualization of networks. *Journal of Complex Networks*, 3(2):159–176, 2014.

[18] Manlio De Domenico, Albert Solé-Ribalta, Emanuele Cozzo, Mikko Kivelä, Yamir Moreno, Mason A Porter, Sergio Gómez, and Alex Arenas. Mathematical formulation of multilayer networks. *Physical Review X*, 3(4):041022, 2013.

[19] Manlio De Domenico, Albert Solé-Ribalta, Sergio Gómez, and Alex Arenas. Navigability of interconnected networks under random failures. *Proceedings of the National Academy of Sciences*, 111(23):8351–8356, 2014.

[20] Manlio De Domenico, Albert Solé-Ribalta, Elisa Omodei, Sergio Gómez, and Alex Arenas. Ranking in interconnected multilayer networks reveals versatile nodes. *Nature communications*, 6, 2015.

[21] Michael C Frank, Mika Braginsky, DANIEL Yurovsky, and Virginia A Marchman. Wordbank: An open repository for developmental vocabulary data. *Journal of child language*, 2016.

[22] Joaquín Goñi, Gonzalo Arrondo, Jorge Sepulcre, Inigo Martincorena, Nieves Vélez de Mendizábal, Bernat Corominas-Murtra, Bartolomé Bejarano, Sergio Ardanza-Trevijano, Herminia Peraita, Dennis P Wall, et al. The semantic organization of the animal category: Evidence from semantic verbal fluency and network theory. *Cognitive processing*, 12(2):183–196, 2011.

[23] Thomas L Griffiths, Mark Steyvers, and Alana Firl. Google and the mind predicting fluency with pagerank. *Psychological Science*, 18(12):1069–1076, 2007.

[24] Geoffrey Grimmett and David Stirzaker. Probability and random processes, 2001.

[25] Thomas T Hills, Mounir Maouene, Josita Maouene, Adam Sheya, and Linda Smith. Longitudinal analysis of early semantic networks preferential attachment or preferential acquisition? *Psychological Science*, 20(6):729–739, 2009.

[26] Ramon Ferrer i Cancho and Richard V Solé. The small world of human language. *Proceedings of the Royal Society of London. Series B: Biological Sciences*, 268(1482):2261–2265, 2001.

[27] Marcus Kaiser. Mean clustering coefficients: the role of isolated nodes and leafs on clustering measures for small-world networks. *New Journal of Physics*, 10(8):083042, 2008.

[28] Mikko Kivelä, Alex Arenas, Marc Barthelemy, James P Gleeson, Yamir Moreno, and Mason A Porter. Multilayer networks. *Journal of Complex Networks*, 2(3):203–271, 2014.

[29] Brian MacWhinney. *The CHILDES project: The database*, volume 2. Psychology Press, 2000.

[30] Ken McRae, George S Cree, Mark S Seidenberg, and Chris McNorgan. Semantic feature production norms for a large set of living and nonliving things. *Behavior research methods*, 37(4):547–559, 2005.

[31] George A Miller. WordNet: a lexical database for english. *Communications of the ACM*, 38(11):39–41, 1995.

[32] Adilson E Motter, Alessandro PS de Moura, Ying-Cheng Lai, and Partha Dasgupta. Topology of the conceptual network of language. *Physical Review E*, 65(6):065102, 2002.

[33] Douglas L Nelson, Cathy L McEvoy, and Thomas A Schreiber. The University of South Florida free association, rhyme, and word fragment norms. *Behavior Research Methods, Instruments, & Computers*, 36(3):402–407, 2004.

[34] Mark Newman. *Networks: an introduction.* Oxford University Press, 2010.

[35] Mariano Sigman and Guillermo A Cecchi. Global organization of the WordNet lexicon. *Proceedings of the National Academy of Sciences*, 99(3):1742–1747, 2002.

[36] Massimo Stella and Markus Brede. Patterns in the English language: Phonological networks, percolation and assembly models. *Journal of Statistical Mechanics*, 2015:P05006, 2015.

[37] Massimo Stella and Markus Brede. Investigating the phonetic organisation of the english language via phonological networks, percolation and Markov models. In *Proceedings of ECCS 2014*, pages 219–229. Springer International Publishing, 2016.

[38] Mark Steyvers and Joshua B Tenenbaum. The large-scale structure of semantic networks: Statistical analyses and a model of semantic growth. *Cognitive science*, 29(1):41–78, 2005.

[39] Michael S Vitevitch. What can graph theory tell us about word learning and lexical retrieval? *Journal of Speech, Language, and Hearing Research*, 51(2):408–422, 2008.

[40] Stanley Wasserman and Katherine Faust. *Social network analysis: Methods and applications*, volume 8. Cambridge university press, 1994.

[41] Duncan J Watts and Steven H Strogatz. Collective dynamics of 'small-world' networks. *nature*, 393(6684):440–442, 1998.

[42] Michael Zock. Words in books, computers and the human mind. *Journal of Cognitive Science*, 16(4):355–378, 2015.
